# Emotion regulation beyond executive and attention difficulties: impact on daily life impairments in community adolescents

**DOI:** 10.1186/s13034-025-00898-1

**Published:** 2025-04-29

**Authors:** Elena Poznyak, Martin Debbané

**Affiliations:** 1 Developmental Clinical Psychology Research Unit, Faculty of Psychology and Educational Sciences, University of Geneva, 40 Boulevard du Pont d’Arve, 1205 Geneva, Switzerland; 2 Research Department of Clinical, Educational and Health Psychology, University College London, London, UK

**Keywords:** Adolescence, ADHD, Emotion regulation, Emotion reactivity, Functional impairment

## Abstract

**Background:**

It is becoming widely recognized that emotion regulation difficulties are an essential feature present along the continuum from subclinical to clinical Attention Deficit/Hyperactivity Disorder (ADHD). Yet, it remains unclear whether and how specific processes related to emotion regulation contribute to daily life impairments, across different domains of functioning. The aim of this cross-sectional study in community adolescents was to investigate whether three processes commonly implicated in adaptive emotion regulation—emotion recognition, emotion reactivity and use of cognitive emotion regulation strategies—uniquely contribute to adolescent-rated functional impairment, above and beyond the effects of age and gender, ADHD symptoms, and individual differences in verbal ability and executive functions.

**Methods:**

161 adolescents from the general population (mean age = 15.57; SD = 1.61) completed the Weiss Functional Impairment Scale, the Emotion Reactivity Scale, the Cognitive Emotion Regulation Questionnaire and the Geneva Emotion Recognition Test. Hierarchical regression analysis examined the unique contributions of candidate predictors to impairment scores.

**Results:**

Total impairment scores were best predicted by older age, inattention symptoms, higher emotion reactivity, and higher use of maladaptive cognitive emotion regulation strategies. Emotion regulation processes were associated with interpersonal difficulties and self-concept impairments, whereas inattention symptoms were associated with school and life skills impairments.

**Conclusions:**

This study stresses that emotion reactivity and maladaptive cognitive emotion regulation represent major sources of perceived social and emotional difficulties in community adolescents. Our results also support the continuum hypothesis of attention difficulties, where emotion regulation abilities may at least partially explain the association between ADHD symptoms and social impairments. Together, these findings highlight the vital importance of targeting emotion regulation in psychotherapeutic interventions aiming to improve socio-emotional outcomes in adolescents.

## Background

Pathways through which Attention Deficit/Hyperactivity Disorder (ADHD) symptoms affect real-life impairments across different domains are still poorly understood, posing obstacles to personalized treatment strategies. Core symptoms of ADHD (inattention and hyperactivity/impulsivity symptoms) typically emerge in childhood, but tend to persist into adolescence causing considerable functional impairment [1, 2]. Functional impairment can be defined as the disruptions in one’s daily functioning and is typically assessed by rating scales evaluating functioning in key areas, such as school/work, family, life skills or social functioning [3]. Studies assessing functional impairment in youths with ADHD demonstrate that compared to controls, they manifest more struggles across all of the aforementioned domains [3]. Importantly, social impairments are of particular concern for adolescents with ADHD: they are often less liked and more rejected by their peers, they struggle to build and maintain positive relationships and have more family conflicts [4–6].

Previous research has identified several candidate factors that may influence outcomes in youths with ADHD. First and foremost, dimensions of inattention and hyperactivity/impulsivity seem to differentially contribute to functional outcomes. Inattention symptoms have been most often associated with school impairments, whereas hyperactivity/impulsivity symptoms have been linked to disruptive behaviours [7, 8]. Both inattention and hyperactivity/impulsivity have been associated with social impairments [7, 9, 10], although the link between ADHD core symptoms and social functioning may not be straightforward. For example, longitudinal studies demonstrate that core ADHD symptoms vary considerably from childhood to late adolescence, whereas social impairments, in contrast, are more persistent across the lifespan [11]. Moreover, one study in a large sample of children and adolescents, showed that after accounting for oppositional defiant symptoms, the contribution of core ADHD symptoms to interpersonal impairment was only minimal [8]. Hence, there is a need to explore how other factors can contribute to social problems in youths with attention difficulties.

Another line of evidence suggests that cognitive impairments sometimes associated with ADHD could represent an important risk factor for poorer outcomes. For instance, some studies link lower global intellectual abilities and lower verbal ability in particular to poorer daily life outcomes [12–15]. The most widely investigated cognitive candidate of impairment in ADHD is without doubt executive function difficulties. Executive functions (EF), including working memory, cognitive flexibility and inhibitory control are higher order, top-down cognitive processes considered critical for regulating emotions and behaviour [16]. Barkley’s influential model of ADHD [17] suggests that individuals with ADHD may particularly struggle with regulating arousal for goal-directed activities due to difficulties in inhibitory control: the ability to suppress automatic responses. Nonetheless, the contribution of EF difficulties to social impairments remains unclear. One longitudinal study in girls with ADHD showed that childhood EF predicted peer acceptance in adolescence [18] and there is also some evidence that EF deficits may mediate the association between ADHD symptoms and social impairment in children [19, 20]. In contrast, other studies found no conclusive associations between EF and social functioning [21–23].

A more recent hypothesis posits that emotion regulation abilities may play a pivotal role in social impairments observed in youths with ADHD, possibly above and beyond the effects of core symptoms of inattention and hyperactivity/impulsivity and associated cognitive impairments [24, 25]. In fact, it is increasingly recognized that emotion regulation difficulties represent and additional marker of ADHD leading to poorer outcomes across the lifespan [26–28]. Examples of emotion regulation difficulties in adolescents with ADHD can include irritability [29], emotional lability [30] and low tolerance to frustration [31], which may have important repercussions on daily life functioning. For example, longitudinal studies show associations between emotion regulation difficulties and poorer academic, occupational and social functioning, as well as increased psychiatric comorbidity later in life [32, 33]. Nevertheless, it remains to be clarified which of the different processes involved in emotion regulation relate to different domains of functioning.

Remarkably, there is currently no clear definition of emotion regulation and no consensus about its exact components, which denotes a major challenge to address them experimentally. R. Thompson defined emotion regulation as the “*extrinsic and intrinsic processes responsible for monitoring*, *evaluating*, *and modifying emotional reactions*, *especially their intensive and temporal features*, *to accomplish one’s goals*” [34]. It is generally assumed that emotion regulation is multidimensional and probably involves different psychological processes operating at different stages of complexity [26, 35]. In the current study, we propose to investigate emotion regulation by looking at three core components: identification of emotions (emotion recognition); impact of emotions on oneself (emotion reactivity) and regulation of emotions through appraisal (cognitive emotion regulation/coping strategies).

Emotion recognition can be seen as one of the first and most basic stages of emotion regulation, that implies the correct detection of emotional cues, typically based on non-verbal information (e.g. voice, facial expression, posture). Emotion recognition ability typically improves in adolescents as they build more complex relationships and become more sensitive to peer evaluations [36, 37]. Accurately detecting emotional cues in others becomes particularly relevant to appropriately respond to social interactions. Accordingly, better emotion recognition abilities were previously linked to better interpersonal outcomes in community adolescents [38]. In contrast, several studies in adolescents with ADHD have demonstrated lower performance on emotion recognition tasks as compared to controls, pointing to difficulties identifying positive and negative emotions, which have been further linked to impairments in social functioning [39–42].

Emotion reactivity refers to the extent to which one experiences emotions: (a) in response to a wide array of stimuli (emotion sensitivity), (b) strongly or intensely (emotion intensity), and (c) for a prolonged period of time before returning to baseline level of arousal (emotion persistence) [43]. Emotion reactivity rapidly increases in early adolescence, most notably with the onset of puberty [44]. Heightened emotion reactivity has been linked to poorer social functioning in healthy adolescents [45]. A meta-analysis of 22 studies that included over 21,000 participants found that youths with ADHD have important difficulties in modulating their reactivity to novel or stressful events [25]. For example, in children and adolescents with ADHD symptoms, intense and rapidly escalating emotional responses have been particularly observed during social rejection tasks [46]. Accordingly, emotion reactivity has been consistently linked to social and emotional problems in ADHD across different age groups [27, 47, 48].

Finally, cognitive emotion regulation can be defined as the use of conscious, cognitive strategies to cope with an emotional experience, in an adaptive (e.g. positive reappraisal, focus on planning, acceptance) or maladaptive way (e.g. blame self or others, catastrophizing, ruminating) [49]. The transition from adolescence to adulthood is typically associated with more efficient, adaptive cognitive emotion regulation, which is also associated with better social functioning [50, 51]. Preliminary evidence shows that compared to controls, adolescents with ADHD can use more maladaptive, and less adaptive cognitive emotion regulation strategies [52]. The associations between adaptive and maladaptive strategies use and social difficulties in daily life merits further scrutiny.

In summary, previous research suggests that emotion recognition, emotion reactivity and cognitive emotion regulation can be altered in adolescents with ADHD and possibly linked to social impairments. However, a major outstanding question is how emotional processes relate to poorer outcomes above and beyond the effects of core ADHD symptoms and variation in EF abilities. Clinical studies often include highly heterogeneous samples with more severe symptoms, increased cognitive and emotional problems, longer trajectories with comorbid disorders and medication effects, which make it challenging to interpret the direct associations between potential predictors and impairment ratings [53]. Importantly, a better characterization of the potential predictors of impairment can arise from studies in the general population. In fact, according to the dimensional view of ADHD [54], the ADHD diagnosis represents the extreme of a continuum of symptoms of inattention and hyperactivity/impulsivity in the general population. Accordingly, associations between ADHD symptoms, emotion regulation difficulties and impairment have also been observed in community samples [55]. More generally, the continuum view of psychopathology has proven extremely useful in identifying risk and protective factors implicated in psychiatric disorders [56, 57]. According to this framework, rather than representing distinct entities, psychopathological symptoms are dimensional in nature and exist on a spectrum, determined by complex interactions between genetic, neurobiological and environmental factors, sometimes leading to full-blown clinical manifestations and sometimes causing only mild alterations in functioning [58]. Thus, using community samples in psychopathology research enables to grasp a broader range of symptoms and associated impairments.

The objective of this study is to elucidate the paths to impairments in different areas of functioning, as they are perceived and rated by adolescents from the general population. Precisely, we sought to explore whether emotion processing factors (emotion recognition, emotion reactivity and cognitive emotion regulation) add any predictive value in explaining daily life impairments above and beyond the effects of ADHD symptoms and executive functions.

Because of the choice to use a self-report measure to assess functional impairment, we included a measure of verbal ability as an additional cognitive variable of interest, as it may affect adolescents’ comprehension and introspection ability. We also aimed to control for the potential effects of age and gender, as they often have important effects on emotion regulation and perceived difficulties in adolescents [59–62].

We hypothesized that processes related to emotion regulation would show distinctive contributions to daily life impairment, beyond the effects of demographics (age and gender), core ADHD symptoms (inattention and hyperactivity-impulsivity) and cognitive factors (verbal reasoning and executive functions). We expected to observe effects of emotional processing factors specifically in the domains reflecting adaptive social functioning, as opposed to academic performance.

## Methods

### Participants

Participants were recruited from the general population as part of a research project investigating associations between attentional and emotional difficulties in adolescence. The project was advertised at the University of Geneva and within the community. Undergraduate students at the Faculty of Psychology also assisted with the recruitment as part of their course requirements. Inclusion criteria were age (12–18), fluency in French and absence of prior or current psychiatric diagnoses or learning disorders.

Amongst 204 subjects who took part in the study, 164 adolescents were selected for the analysis as they completed both the Weiss Functional Impairment Scale (WFIRS-S) and the ADHD Self-Report Scale (ASRS v1.1). Two participants were excluded as they obtained WISC-V Vocabulary scores below 5, which could suggest some learning difficulties. One additional participant was excluded as they had a diagnosis of dyslexia. The final sample comprised 161 adolescents (103 females, 58 males, age range 12–18, mean age = 15.57; SD = 1.61). All were enrolled in a school program at the time of the study (compulsory school for younger subjects and high school or professional training for older adolescents).

## Measures

### Functional impairment

The French version of the Weiss Functional Impairment Scale Self-report (WFIRS-S) was used to assess functional impairment [63]. This self-report instrument has demonstrated good reliability and validity in assessing functional impairment in both clinical and non-clinical populations [3].

The WFIRS-S consists of a total of 69 items assessing impairment in seven domains of functioning including Family (8 items), Work (11 items), School (10 items), Life skills (12 items), Self- Concept (5 items), Social activities (9 items) and Risky activities (14 items). Previous studies using the WFIRS-S in adolescents and emerging adults have demonstrated good to excellent internal consistency for the total score and subscales, with Chronbach’s alpha values ranging from 0.72 to 0.94. [64, 65]. The items are rated on a 0–3 point Likert scale (never or not at all (0), sometimes or a little (1), often or moderately (2), very often or a lot (3)). Participants also had the option to rate an item as non-applicable. The Work domain was not used for the analysis as most adolescents had no professional activity at the time of the study. For the remaining six domains, a mean subscale score was computed as the total score per domain divided by the number of items answered in each domain. Clinical guidelines suggest that any domain with a mean score above 1.5, two items with a score equal or above 2, or one item with a score equal to 3 can be considered as impaired. A total impairment score was also calculated as the sum of scores on all items answered across all six domains. For both mean scores and the total score, higher values indicate greater impairment.

### ADHD symptoms

The ADHD Self-Report Scale (ASRS v1.1) was used to assess symptoms of Attention-Deficit/Hyperactivity Disorder (ADHD). The ASRS is a widely used self-report questionnaire developed by the World Health Organization (WHO) [66], that showed good reliability and validity in adolescent samples [67, 68]. It consists of 18 items that capture symptoms related to both inattention (9 items) and hyperactivity/impulsivity (9 items), aligning with the criteria outlined in the Diagnostic and Statistical Manual of Mental Disorders (DSM-5). Participants rate the frequency of experiencing each symptom over the past six months on a 0–4 Likert scale (0 = never; 1 = rarely; 2 = sometimes; 3 = often; 4 = very often). Item scores are summed to obtain total scores for inattention and hyperactivity/impulsivity, which were used for the analysis.

### Cognitive functioning

#### Verbal reasoning

The Vocabulary subtest of the Wechsler Intelligence Scale for Children (WISC-V) or Adults (WAIS-IV) was used to assess general verbal ability. The subtest from the WAIS-IV was used for participants over 16 years of age and the subtest from the WISC-V was used for participants below 16 years of age. The Vocabulary subtest is designed to assess the participant’s word knowledge and verbal concept formation abilities. The subtest involves presenting participants with a series of words that they are required to define. These words increase in difficulty as the test progresses. Participants’ responses are scored using a standardized scoring system provided in the test manual, with a maximum of 2 points attributed for each item. The total raw score obtained is converted to a scaled score (with a mean of 10 and standard deviation of 3), based on normative data. The scaled score was used for the analysis.

#### Executive functions

The visual Go No-Go task, also known as the Sustained Attention to Response Task (SART) [69], was used to assess sustained attention and the ability to inhibit prepotent responses. The Go No-Go task is a classic experimental paradigm wherein participants are presented with a series of stimuli and instructed to respond selectively to one type of stimulus (Go trials) while withholding responses to another type (No-Go trials). In our implementation, participants were presented with a rapid sequence of 225 numbers (from 1 to 9), on a computer screen. Each number was presented for 250 ms and followed by a 900 ms visual mask. There was a 1150 ms interval between stimuli. The entire task had a duration of 4.3 min. Participants were instructed to press the Space bar on the keyboard as quickly as possible in response to the target stimulus (number “3”) while refraining from responding to other numbers. The percentage of omission and inhibition errors were used for the analysis.

### Emotional processing variables

#### Emotion recognition

The Geneva Emotion Recognition Test -Short version (GERT-S) [70] was used to assess participants’ abilities in recognizing facial expressions of emotion. The GERT-S is a non-verbal computerized task. It presents participants with 42 short videos with sound (duration 1–3 s), depicting male and female actors expressing 14 different emotions (joy, sadness, anger, fear, disgust, surprise, anxiety, amusement, pride, pleasure, relief, interest, irritation, despair). Thus, the test encompasses both basic and more nuanced emotions, of different arousal and valence levels. Participants are asked to identify the emotion expressed in each video sequence from a list of 14 emotions. Each emotion is presented 3 times in the task. Each correct identification earns one correct answer, providing a total accuracy score (/42). The GERT-S has demonstrated good reliability as a brief measure of general emotion recognition with a unidimensional structure. The total accuracy score was used for the analysis.

#### Emotion reactivity

The Emotion Reactivity Scale (ERS), French validated version [71] is a self-report questionnaire designed to assess individual differences in emotion reactivity, based on three aspects: intensity, duration, and persistence of emotional responses. It consists of 21 items that prompt participants to reflect on their typical reactions to emotionally evocative situations, such as feeling upset, excited, or anxious. Participants rate the extent to which each statement describes their emotional experiences using a 5-point Likert scale from 0 (not at all like me) to 4 (completely like me). The total score, with a maximum possible score of 84, was used for the analysis.

#### Cognitive emotion regulation

The Cognitive Emotion Regulation Questionnaire (CERQ) French validated version [72] was used to assess emotion regulation. The CERQ is a self-report instrument originally validated in adolescents [49] and designed to evaluate cognitive coping strategies individuals employ in response to stressful or emotionally challenging situations. It consists of 36 items that capture nine cognitive emotion regulation strategies identified through principal component analysis. Five strategies are considered as adaptive, including acceptance (e.g. having thoughts of acceptance and resignation towards the situation), positive refocusing (e.g. having thoughts that are positive and pleasant instead of thinking about the negative situation), refocus on planning (e.g. having thoughts about what to do to handle the situation), positive reappraisal (e.g. thinking about giving a positive meaning to the situation in terms of personal growth) and putting into perspective (e.g. having thoughts that relativize the situation compared to other experiences). The remaining four strategies are considered as maladaptive, including rumination (thinking about thoughts and feelings associated with the negative events), catastrophizing (thinking about the how negative and terrible the experience was), self-blame (e.g. having thoughts that blame oneself for what has happened) and blaming others (e.g. having thoughts that blame others for what has happened). Participants rate the frequency with which they engage in each strategy when facing difficult emotions using a 5-point Likert scale ranging from 1 (almost never) to 5 (almost always). For the analysis, mean scores were computed for each subscale, reflecting adaptive strategies use and maladaptive strategies use.

### Procedure

All participants agreed to participate in the study on a voluntary basis and written consent was obtained from adolescent’s parents. Participants were invited for an experimental session led by a trained research team member (master or doctoral level psychology student). The experimental session lasted approximately 90–120 min and comprised a series of cognitive and experimental tasks, including the WAIS/WISC Vocabulary subtest, the Go No Go task and the GERT. Due to the COVID-19 pandemic, the experimental sessions were conducted in hybrid mode, sometimes in person and sometimes by video call. In addition, participants were asked to complete a series of self-report questionnaires online, including the WFIRS-S, the ASRS v.1.1, the ERS and the CERQ.

### Statistical analysis

All analysis were performed with SPSS V.29 for Mac OS. Data distribution was visually inspected for all variables of interest. Extreme values were removed and treated as missing data. Missing data was dealt with multiple imputation with means replacement.

First, the associations between demographic characteristics and scores on all tests and questionnaires were explored. As our sample was predominantly female, independent samples tests (Welch t-test for unequal sample sizes) were used to examine gender differences on all measures. Associations between age and all variables were performed using Pearson’s correlations.

Then, hierarchical linear regression models were built to explore associations between the hypothesized predictors and the total WFIRS-S score. The aim of this analysis was to investigate the additive effects of demographic, ADHD symptoms, cognitive and emotional variables on functional impairment scores. All assumptions for conducting linear regressions were met. Normality and homoscedasticity were verified with skewness values (< 2), P-P plots and scatterplots of residuals, confirming no significant deviations. Multicollinearity was checked using Variance Inflation Factor (VIF) and tolerance values which showed no multicollinearity between independent variables (VIF < 10; tolerance > 0.1).

Continuous independent variables were centered around the mean. We first defined a baseline model (Model 0) with only age and gender as predictors of the dependent variable.

Then, in Step 1 of the analysis, we included ADHD symptoms (ASRS v.1.1 inattention and hyperactivity/impulsivity scores). In step 2, we added cognitive functioning variables (verbal reasoning, assessed by the WISC/WAIS Vocabulary and attention and executive functioning, assessed by the Go No Go omission and inhibition errors, respectively). Finally, in step 3, we entered emotional processing variables (GERT emotion recognition, CERQ adaptive and maladaptive emotion regulation, ERS emotion reactivity) to explore whether they add any predictive value to the model, above and beyond the variance explained by age and gender, ADHD symptoms, and cognitive factors.

The same linear regression models were conducted on the WFIRS-S subscales, in a post-hoc fashion, in order to examine possible differential associations between selected predictors and different domains of functioning.

## Results

### Age and gender differences

Descriptive statistics and gender differences on all administered measures are presented in Table 1. Gender differences were observed on most variables of interest. There was a small, but statistically significant age difference observed between girls (*M* = 15.79, *SD* = 1.56) and boys (*M* = 15.17, *SD* = 1.65). Overall, girls reported more functional impairment across all domains except Risky activities which did not differ by gender. Girls also reported more ADHD symptoms, a higher use of maladaptive emotion regulation strategies and higher emotion reactivity scores. In contrast, girls showed better emotion recognition scores and committed less omission and inhibition errors on the EF task.

Older age was associated with better attentional performance (less omission *r*=-.285, *p* <.001 and inhibition errors *r* = −.249, *p* =.001) and better emotion recognition on the GERT (*r* =.322, *p* <.001). Older adolescents also tended to have higher total functional impairment scores (*r* =.215, *p* =.006), and more specifically higher impairment ratings in the Self-Concept domain (*r* =.249, *p* =.001).

**Table 1:** Descriptive statistics and gender differences on all variables

	Females (*n* = 103)	Males (*n* = 58)	Statistic	*p*
Age (years)	15.79 (1.56)	15.17(1.65)	5.55	.020
WISC/WAIS Vocabulary (scaled score)	12.09 (2.76)	12.14 (2.69)	0.01	.910
**Functional impairment (WFIRS-S)**				
Total score	36.20 (20.39)	24.57(15.75)	16.28	<.001
Family	0.96 (0.59)	0.65(0.51)	12.19	<.001
School	0.68 (0.46)	0.51(0.49)	4.59	.034
Life Skills	0.73(0.49)	0.54(0.48)	6.13	.015
Self-Concept	1.36 (0.85)	0.80(0.78)	17.27	<.001
Social Activities	0.65 (0.46)	0.44(0.36)	10.16	.002
Risky Activities	0.31 (0.39)	0.32 (0.30)	0.08	.775
**ADHD Symptoms (ASRS v1.1)**				
Inattention (/36)	19.56 (5.62)	16.83 (5.50)	9.02	.003
Hyperactivity/Impulsivity (/36)	15.89 (5.46)	14.21 (4.47)	4.48	.036
**Cognitive Emotion Regulation Questionnaire (CERQ)**				
CERQ Adaptive strategies use	12.75 (2.51)	13.05 (2.68)	0.50	.481
CERQ Maladaptive strategies use	10.55 (2.48)	9.31 (2.10)	11.33	<.001
**Emotion Reactivity Scale (ERS)**				
ERS Total Score (/84)	38.91 (16.76)	27.44 (15.99)	18.42	<.001
**Emotion Recognition (GERT-S)**				
GERT Total Score (/42)	26.49 (4.53)	24.43 (5.17)	6.37	.013
**Executive function (Go No Go)**				
Omission errors (%)	3.82 (4.92)	6.32(6.44)	6.60	.012
Inhibition errors (%)	51.73 (19.66)	65.16(20.03)	16.90	<.001

The Welch t-test was used to examine effects of gender. Significance was established at *p* <.05

### Predictors of total WFIRS-S scores

Model statistics are presented in Table 2. The baseline model showed that older age and female gender were associated with higher impairment scores.

Model 1, which included ASRS inattention and hyperactivity/impulsivity scores explained an additional 20.5% of variance in impairment scores.

Model 2, including the cognitive variables showed no significant improvement from Model 2.

Model 3 however showed substantial improvement in the prediction of total impairment scores, explaining overall 47.1% of variance in the dependent variable. Higher emotional reactivity and higher use of maladaptive emotion regulation strategies emerged as significant predictors of higher functional impairment, alongside inattention symptoms and age. We note that the main effects of gender and of hyperactivity/impulsivity were no longer significant after the inclusion of variables reflecting emotional processing.

**Table 2:** Summary of the Hierarchical Multiple Regression Analysis on the Weiss Functional Impairment Scale Total Score

Independent variable	B	SE	Beta	*p*	*R*²	ΔR²	F Change	Sig F change
**Model 0 Demographic Factors**					0.109	0.109	9.642	<.001
Age	2.039	0.928	0.168	.030				
Gender (0 = male, 1 = female)	10.360	3.114	0.254	.001				
**Model 1 ADHD Symptoms**					0.313	0.205	23.258	<.001
Age	1.759	0.825	0.145	.035				
Gender 0 = male 1 = female	6.093	2.822	0.150	.032				
ASRS Inattention	1.109	0.261	0.323	<.001				
ASRS Hyperactivity-Impulsivity	0.836	0.283	0.221	.004				
**Model 2 Cognitive Factors**					0.338	0.024	1.871	.137
Age	1.945	0.849	0.160	.023				
Gender	7.577	2.955	0.186	.011				
ASRS Inattention	1.112	0.260	0.324	<.001				
ASRS Hyperactivity-Impulsivity	0.694	0.290	0.183	.018				
WISC-WAIS Vocabulary	0.952	0.497	0.132	.057				
GnG Omission errors	0.055	0.271	0.016	.839				
GnG Inhibition errors	0.088	0.073	0.093	.229				
**Model 3 Emotional Processing**					0.471	0.133	9.399	<.001
Age	1.872	0.809	0.154	.022				
Gender	3.443	2.777	0.085	.217				
ASRS Innatention	0.760	0.244	0.222	.002				
ASRS Hyperactivity-Impulsivity	0.367	0.271	0.097	.178				
WISC-WAIS Vocabulary	0.632	0.472	0.088	.183				
GnG Omission errors	0.172	0.255	0.049	.502				
GNG Inhibition errors	0.089	0.069	0.094	.196				
GERT	0.489	0.284	0.121	.087				
ERS	0.202	0.092	0.178	.030				
CERQ adaptive strategies	−0.543	0.488	−0.071	.268				
CERQ maladaptive strategies	2.014	0.623	0.249	.002				

**B**: Unstandardized regression coefficient; **SE**: Standard error of B; **Beta**: Standardized regression coefficient; **R²**: R-squared value indicating the proportion of variance explained by the model; **ΔR²**: Change in R-squared from the previous step; **F Change**: F-statistic for the change in R-squared; **Sig. F Change**: Significance level of the F change.

Significance level was set at *p* <.05.

### Predictors of impairment by WFIRS-S domains

#### Family functioning

Model 0 with demographic variables retained female gender as a significant predictor of impairment (*B* = 0.321, *SE* = 0.095, *p* <.001), but was weak in terms of amount of variance explained (*∆F* (2,160) = 5.735, *p* =.004, *R*^*2*^ = 0.068). Model 1 (*∆F* (2,156) = 13.739, *p <*.001, *∆R*^*2*^ = 0.140) revealed additional significant effects of inattention (*B* = 0.025, *SE* = 0.008, *p* =.003) and hyperactivity/impulsivity symptoms (*B* = 0.023, *SE* = 0.009, *p* =.01). Model 2 did not result in significant change. Model 3 (∆*F* (4,150) = 5.601, *p <*.001, *∆R*^*2*^ = 0.102) identified higher use of maladaptive emotion regulation strategies as the strongest predictor of Family impairment (*B* = 0.054, *SE* = 0.021, *p* =.01), whereas the effects of gender and hyperactivity/impulsivity disappeared and the effect of inattention remained marginally significant (*p* =.04).

#### School functioning

Model 0 showed no significant effects of age and gender. The addition of ADHD symptoms significantly improved the model (∆*F* (2,156) = 20.467, *p <*.001, *∆R*^*2*^ = 0.240). Inattention symptoms showed to be the strongest correlate of impairment (*B* = 0.032, *SE* = 0.07, *p* <.001), whereas the effect hyperactivity/impulsivity did not reach significance (*p* =.07). The addition of the effects of cognitive and emotional processing variables in steps 2–3 did not significantly improve model predictions.

#### Self-concept

The baseline Model 0 was significant: older age (*B* = 0.107, *SE* = 0.041, *p* =.009) and female gender (*B* = 0.486, *SE* = 0.136, *p* <.001) were associated with higher impairment (F(2, 160) = 12.03, *p* <.001; *R*^*2*^ = 0.132). Model 1(∆*F* (2,156) = 9.514, *p <*.001, ∆*R*^*2*^ = 0.094), showed additional effects of Inattention (*B* = 0.025, *SE* = 0.012, *p* =.04) and Hyperactivity/Impulsivity (*B* = 0.035, *SE* = 0.013, *p* =.010), whereas step 2 did not yield significant change.

Model 3 accounted for an additional 21.3% of variance (∆*F*(2,150) = 14.507, *p <*.001, *∆R*^*2*^ = 0.213) and retained older age, (*B* = 0.080, *SE* = 0.036, *p* =.030), higher emotional reactivity (*B* = 0.010, *SE* = 0.004, *p* =.019), higher use of maladaptive emotion regulation (*B* = 0.112, *SE* = 0.028, *p* <.001) and better emotion recognition scores (*B* = 0.038, *SE* = 0.013, *p* =.003) as significant predictors of self- concept impairment. Importantly, the effects of gender and ADHD symptoms were no longer significant.

#### Social activities

The baseline model (∆*F* (2, 158) = 4.416, *p* =.014, *R*^*2*^ = 0.054) showed a significant effect of female gender (*B* = 0.208, *SE* = 0.072, *p* =.005). Model 1 (∆*F*(2,154) = 6.309, *p* =.002, *∆R*^*2*^ = 0.072) added a significant effect of hyperactivity/impulsivity symptoms (*B* = 0.018, *SE* = 0.007, *p* =.014). However, the effects of hyperactivity/impulsivity disappeared in Model 2 (∆*F*(3,151) = 3.864, *p* =.011, ∆*R*^*2*^ = 0.062), which instead highlighted the effects of Vocabulary (*B* = 0.040, *SE* = 0.012, *p* =.001) and female gender. Finally, the addition of emotional variables in Model 3 (∆*F* (4,147) = 6.281, *p* <.001) identified higher Vocabulary scores (*B* = 0.032, *SE* = 0.012, *p* =.01) and higher use of maladaptive emotion regulation strategies (*B* = 0.063, *SE* = 0.016, *p* <.001) as best predictors of social problems, whereas the effects of gender and hyperactivity/impulsivity were no longer significant.

#### Life skills

The baseline model (∆*F*(2, 160) = 4.04, *p* =.019, *R*^*2*^ = 0.049) showed a significant effect of female gender on impairment (*B* = 0.175, *SE* = 0.081, *p* =.032). Model 1 highly improved the baseline model (∆*F*(2,156) = 17.708, *p* <.001, ∆*R*^*2*^ = 0.176) by adding significant effects of inattention (*B* = 0.028, *SE* = 0.007, *p* <.001) and hyperactivity/impulsivity (*B* = 0.017, *SE* = 0.008, *p* =.008). Model 2 resulted in a small but significant improvement (∆*F*(2,154) = 3.455, *p* =.034, ∆*R*^*2*^ = 0.033): a significant effect of Vocabulary emerged (*B* = 0.032, *SE* = 0.013, *p* =.017) and the effects of gender and hyperactivity/impulsivity disappeared.

In the final Model 3 (∆*F*(4,150) = 3.802, *p* =.006, ∆*R*^*2*^ = 0.068), inattention remained as the only strongest correlate of impairment. There was also a marginal effect of maladaptive emotion regulation strategies (*p* =.05).

#### Risky activities

All models were weak in terms of explanatory power and there were no significant associations between any of the candidate predictors and impairment scores in this domain.

## Discussion

This study in community adolescents sought to test the hypothesis that processes implicated in emotion regulation (emotion recognition, emotion reactivity and use of cognitive emotion regulation strategies) contribute to adolescent-rated functional impairment, above and beyond the effects of age and gender, ADHD symptoms, and individual differences in verbal ability and executive functions. The main results identified older age, inattention symptoms, higher emotion reactivity, and higher use of maladaptive cognitive emotion regulation strategies as significant predictors of adolescent-reported total impairment scores. In agreement with our initial hypothesis, emotion regulation processes showed unique contributions to interpersonal difficulties and self-concept impairment, whereas inattention symptoms were more strongly associated with school and life skills impairments. These results confirm that emotion regulation difficulties represent a major source of impairment in adolescents and that this association is observable not only in clinical populations, but also in community samples [60, 73]. Crucially, our findings also provide additional support for the postulate that emotion regulation difficulties may play a detrimental role in explaining the well-documented association between ADHD symptoms and daily life impairment [42, 48].

Regarding the roles of ADHD symptoms, higher inattention scores were particularly associated with impairment in school and life skills, and to a lesser extent—family domains. Our findings replicate existing evidence from both cross-sectional [7, 8] and longitudinal studies [10] that recognize inattention as the strongest predictor of school impairment in children and adolescents on the continuum of ADHD symptoms. Symptoms such as careless mistakes, forgetfulness, distractibility, and poor organizational skills may particularly alter school functioning but can also contribute to reduced autonomy in daily activities (e.g. forgetting appointments, managing finances and chores) and in relationships (e.g. losing track of conversations, lead to misunderstandings). Interestingly, there were no conclusive associations between hyperactivity/impulsivity symptoms and impairment in our sample. Effects of hyperactivity/impulsivity were observed on total impairments ratings as well as on several subscales. However, these effects disappeared after the inclusion of emotion regulation variables, which could speak in favor of a potential mediation effect of emotional processes in the association between hyperactivity/impulsivity symptoms and impairment. This pattern of results would highlight the affective component in the symptoms of hyperactivity and impulsivity [74] and underline the importance to target emotion regulation abilities in the attempt to reduce the impact of such symptoms [75].

The main strength of this study lied in the simultaneous exploration of different processes involved in emotion regulation and in the examination of their individual contributions to different domains of impairment. Results revealed that all three emotion regulation processes investigated were uniquely and differentially associated with impairment reflecting negative self-concept and interpersonal functioning, after controlling for commonly recognized confounding variables. Therefore, our results provide additional support for the hypothesis that the effects of emotion regulation difficulties on impairment appear to go beyond the effects of ADHD symptoms and cognitive processes.

Indeed, higher self-reported emotion reactivity was particularly associated with higher impairment scores in the Self-Concept domain, in which most items denote a negative perception of self (e.g. feeling bad about yourself, feeling frustrated with yourself, feeling incompetent). Thus, a poor modulation of immediate affective responses during arousal-evoking events seemed to be particularly related to adolescent’s negative self-image. This supports empirical evidence that adolescents who display heightened emotional responses may be more at risk at developing poor self-esteem as well as internalizing symptoms, including anxiety and depression [76]. On the other hand, increased use of maladaptive cognitive emotion regulation strategies to cope with negative emotional experiences, predicted impairments in the domains of Family, Social Problems and Self-Concept. Hence, difficulties coping with and reappraising negative emotional experiences may have more negative repercussions on interpersonal relationships in adolescents, whether it is with family or peers. Indeed, research in both community and clinical samples has consistently linked maladaptive strategies such as catastrophizing, and self- or other- blame to impairments in social functioning, including relationship difficulties and a negative self-image [77, 78]. Our results also go in line with emerging evidence in ADHD groups, which suggests that difficulties modulating and coping with own’s emotions can exacerbate interpersonal problems in adolescents and young adults with ADHD [48, 79].

Finally, and somewhat surprisingly, better emotion recognition scores were associated with higher impairment rates in the self-concept domain. This goes against evidence that better emotion recognition leads to better social functioning [41]. Potential mechanisms underlying this association are uncertain. This association could reflect socio-cognitive maturation and suggest that adolescents with better ability to identify emotions in others also may have more insight into their own emotional difficulties and thus may manifest higher self-concept scores on self-report. Similarly, a recent study found that adolescents with better emotion recognition ability may experience higher levels of criticism and judgment towards themselves [80]. Alternatively, considering increased sensitivity to others’ emotions and opinions in adolescence, it could reflect a hypervigilance effect resulting from a selective attention bias towards a “social threat”, which is often observed in individuals with both clinical and non-clinical anxiety [81, 82]. However, this bias was probably specific to girls in our sample, given that they performed significantly better on the emotion recognition task, and also reported more emotional difficulties across different self-report measures.

Indeed, our descriptive results highlighted significant age and gender differences on both self-report questionnaires and on experimental tasks, which must be taken into account when interpreting the main findings. For instance, older adolescents reported higher functional impairment scores, which in our community sample was particularly observed in the Self-Concept domain. This finding aligns with another report that used the WFIRS-S in a community sample of adolescents [83]. Similarly, a recent clinical study demonstrated that compared to children, adolescents with ADHD demonstrated poorer daily life outcomes [84]. Higher impairment scores in older adolescents may reflect, on one hand, increased academic and social pressures during this developmental period, and on the other hand, greater self-awareness [85]. Besides, compared to boys, girls reported higher levels of emotion regulation difficulties and functional impairment. Previous research in non-clinical samples supports our findings, highlighting lower self-concept and more internalizing difficulties in female adolescents [60, 65, 86]. This result can be paralleled with gender differences in psychosocial functioning during adolescence: for instance, girls tend to place greater emphasis on relationships and emotional experiences [87]. While this heightened relational and emotional sensitivity can confer advantages—such as stronger empathy, interpersonal awareness, and theory of mind [88–90]—it may also increase vulnerability to social stress and to social comparisons [91]. These pressures can, in turn, contribute to more perceived social and emotional difficulties [60]. Another interpretation of our findings is that girls, could have displayed increased insight into their functioning and potential difficulties. Supporting this interpretation, adolescent girls in our sample outperformed boys on objective experimental tasks assessing inhibition and emotion recognition, suggesting a possible female advantage in both executive functioning and social cognition [89, 92], which in turn could explain increased introspective ability [93]. Nevertheless, our results may be biased by a predominantly female sample. Hence, the reported gender differences must be replicated in future studies with more balanced gender distributions.

Another important finding is that objective measures of executive functions were not associated with adolescent-rated impairment. This result must be replicated in clinical populations, as perhaps the individual differences in EF were not large enough in our community sample. Nevertheless, this stresses that emotional, and not cognitive factors may bear the most weight on perceived daily difficulties in adolescents. Of course, it is well-documented that the development of emotion regulation abilities is intertwined with the development of executive functions in adolescence, accompanied by the maturation of the prefrontal cortex [94]. Thus, it is important to distinguish between objective methods for measuring cognitive and emotional development, such as neuroimaging methods and neuropsychological tasks, and adolescents’ lived experiences and perceived difficulties, which are typically assessed through self-report measures [95]. Both are imperative to grasp psychological mechanisms that contribute to adolescents’ well-being in daily life.

## Limitations and future directions

This was an exploratory cross-sectional study in community adolescents that had some limitations. For example, our results point to some interactions between independent variables and potential mediation and moderation effects would merit further investigation. More specifically, we observed that the effects of core symptoms disappeared or weakened after the inclusion of emotion processing variables. This could point to a hypothesis that emotion regulation can act as a mediator between ADHD symptoms and impairment, and should be researched further. Given the significant gender differences found in most variables, it would also be interesting to examine whether gender moderates the documented associations, ideally in a more gender-balanced sample.

Another limitation was the cross-sectional design of this study. Future prospective studies are needed to clarify how the identified risk factor may affect long-term impairments in different domains. Besides, longitudinal studies can be extremely helpful in elucidating the interactions between the fluctuations in ADHD symptoms and the intertwined development of emotion regulation and executive functions. Our results reflect associations observed in the general population of adolescents and should be interpreted with caution when considering their implications for understanding clinical ADHD. Replicating these analyses in adolescents with clinical ADHD would be important to confirm and extend our findings. Finally, given considerable discrepancies between adolescent- and parent- rated impairments [96] it would be interesting to explore and contrast correlates of impairment based on ratings from different informants, both in community and in clinical samples.

## Conclusions

This cross-sectional study in community adolescents demonstrated that emotion recognition, emotion reactivity and cognitive emotion regulation uniquely and differentially contribute to adolescent-reported social and self-concept problems, above and beyond the effects of attention problems and executive functions. Our results may have important clinical implications, reinforcing the need to address emotion regulation abilities in psychological interventions for adolescents, whether it is to promote resilience in typically developing youths or to address socio-emotional problems in adolescents with ADHD or other clinical conditions.

## Data Availability

The datasets used and/or analyzed during the current study are available from the corresponding author on reasonable request.

## Abbreviations

ADHD: Attention Deficit/Hyperactivity Disorder
WFIRS-S: Weiss Functional Impairment Scale-Self Report
WFIRS-P: Weiss Functional Impairment Scale-Parent Report
CADDRA: Canadian ADHD Resource Alliance
ASRS: ADHD Self-Report Scale
WISC-V: Wechsler Intelligence Scale for Children 5th version
WAIS-IV: Wechsler Intelligence Scale for Adults 4th version
SART: Sustained Attention to Response Task
GERT-S: Geneva Emotion Recognition Test Short version
ERS: Emotion Reactivity Scale
CERQ: Cognitive Emotion Regulation Questionnaire
SPSS: Statistical Package for the Social Sciences

## References

[1] Sibley MH, Arnold LE, Swanson JM, Hechtman LT, Kennedy TM, Owens E, et al. Variable patterns of remission from ADHD in the multimodal treatment study of ADHD. Am J Psychiatry. 2022;179(2):142–51. 34384227 10.1176/appi.ajp.2021.21010032 PMC8810708

[2] Biederman J, Petty CR, Clarke A, Lomedico A, Faraone SV. Predictors of persistent ADHD: an 11-year follow-up study. J Psychiatr Res. 2011;45(2):150–5. 20656298 10.1016/j.jpsychires.2010.06.009 PMC3068747

[3] Weiss MD, McBride NM, Craig S, Jensen P. Conceptual review of measuring functional impairment: findings from the Weiss Functional Impairment Rating Scale. BMJ Ment Health. 2018;21(4):155–64. 10.1136/ebmental-2018-300025 PMC6241626 30314990

[4] Morris S, Sheen J, Ling M, Foley D, Sciberras E. Interventions for adolescents with ADHD to improve peer social functioning: a systematic review and meta-analysis. J Atten Disord. 2021;25(10):1479–96. 32131667 10.1177/1087054720906514

[5] McQuade JD, Hoza B. Peer problems in attention deficit hyperactivity disorder: Current status and future directions. Dev Disab Res Rev. 2008;14(4):320–4. 10.1002/ddrr.35 19072753

[6] Lee Y-c, Yang H-J, Chen VC-h, Lee W-T, Teng M-J, Lin C-H, et al. Meta-analysis of quality of life in children and adolescents with ADHD: By both parent proxy-report and child self-report using PedsQL™. Res Dev Disab 2016;51:160 − 72. 10.1016/j.ridd.2015.11.009 26829402

[7] Meyer J, Alaie I, Ramklint M, Isaksson J. Associated predictors of functional impairment among adolescents with ADHD—a cross-sectional study. Child Adolesc Psychiatry Mental Health. 2022;16(1):29. 10.1186/s13034-022-00463-0 PMC8985377 35382854

[8] Garner AA, O’Connor BC, Narad ME, Tamm L, Simon J, Epstein JN. The relationship between ADHD symptom dimensions, clinical correlates, and functional impairments. J Dev Behav Pediat. 2013;34(7):469–77. 10.1097/DBP.0b013e3182a39890 PMC4014057 24042078

[9] Zoromski AK, Owens JS, Evans SW, Brady CE. Identifying ADHD symptoms most associated with impairment in early childhood, middle childhood, and adolescence using teacher report. J Abnormal Child Psychol. 2015;43:1243–55. 10.1007/s10802-015-0017-8 25899878

[10] Leopold DR, Christopher ME, Olson RK, Petrill SA, Willcutt EG. Invariance of ADHD symptoms across sex and age: A latent analysis of ADHD and impairment ratings from early childhood into adolescence. J Abnormal Child Psychol. 2019;47:21–34. 10.1007/s10802-018-0434-6 PMC6202270 29691720

[11] Franke B, Michelini G, Asherson P, Banaschewski T, Bilbow A, Buitelaar JK, et al. Live fast, die young? A review on the developmental trajectories of ADHD across the lifespan. Eur Neuropsychopharmacol. 2018;28(10):1059–88. 30195575 10.1016/j.euroneuro.2018.08.001 PMC6379245

[12] Ramos-Olazagasti MA, Castellanos FX, Mannuzza S, Klein RG. Predicting the adult functional outcomes of boys with ADHD 33 years later. J Am Acad Child Adolesc Psychiatry. 2018;57(8):571–82. 30071978 10.1016/j.jaac.2018.04.015 PMC6126351

[13] Baggio S, Hasler R, Deiber M-P, Heller P, Buadze A, Giacomini V, et al. Associations of executive and functional outcomes with full-score intellectual quotient among ADHD adults. Psychiatry Res. 2020;294:113521. 33161177 10.1016/j.psychres.2020.113521

[14] Cheung CH, Rijdijk F, McLoughlin G, Faraone SV, Asherson P, Kuntsi J. Childhood predictors of adolescent and young adult outcome in ADHD. J Psychiatr Res. 2015;62:92–100. 25680235 10.1016/j.jpsychires.2015.01.011 PMC4480336

[15] Conti-Ramsden G, Mok PL, Pickles A, Durkin K. Adolescents with a history of specific language impairment (SLI): strengths and difficulties in social, emotional and behavioral functioning. Res Dev Disab. 2013;34(11):4161–9. 10.1016/j.ridd.2013.08.043 PMC3830176 24077068

[16] Diamond A. Executive functions. Ann Rev Psychol. 2013;64(1):135–68. 23020641 10.1146/annurev-psych-113011-143750 PMC4084861

[17] Barkley RA. Behavioral inhibition, sustained attention, and executive functions: constructing a unifying theory of ADHD. Psychol Bull. 1997;121(1):65. 9000892 10.1037/0033-2909.121.1.65

[18] Miller M, Hinshaw SP. Does childhood executive function predict adolescent functional outcomes in girls with ADHD? J Abnormal Child Psychol. 2010;38:315–26. 10.1007/s10802-009-9369-2 PMC2839522 19960365

[19] Berenguer Forner C, Roselló Miranda B, Baixauli Fortea I, Garcia Castellar R, Colomer Diago C, Miranda Casas A. ADHD Symptoms and peer problems: Mediation of executive function and theory of mind. Psicothema. 2017;8:63. 10.7334/psicothema2016.376 29048312

[20] Motamedi M, Bierman K, Huang-Pollock CL. Rejection reactivity, executive function skills, and social adjustment problems of inattentive and hyperactive kindergarteners. Soc Dev. 2016;25(2):322–39. 27158194 10.1111/sode.12143 PMC4856066

[21] Huang-Pollock CL, Mikami AY, Pfiffner L, McBurnett K. Can executive functions explain the relationship between attention deficit hyperactivity disorder and social adjustment? J Abnormal Child Psychol. 2009;37:679–91. 10.1007/s10802-009-9302-8 19184400

[22] Diamantopoulou S, Rydell A-M, Thorell LB, Bohlin G. Impact of executive functioning and symptoms of attention deficit hyperactivity disorder on children’s peer relations and school performance. Dev Neuropsychol. 2007;32(1):521–42. 17650992 10.1080/87565640701360981

[23] Tamm L, Loren RE, Peugh J, Ciesielski HA. The association of executive functioning with academic, behavior, and social performance ratings in children with ADHD. J Learn Disab. 2021;54(2):124–38. 10.1177/0022219420961338 32996376

[24] Shaw P, Stringaris A, Nigg J, Leibenluft E. Emotion dysregulation in attention deficit hyperactivity disorder. Am J Psychiatry. 2014;171(3):276–93. 24480998 10.1176/appi.ajp.2013.13070966 PMC4282137

[25] Graziano PA, Garcia A. Attention-deficit hyperactivity disorder and children’s emotion dysregulation: a meta-analysis. Clin Psychol Rev. 2016;46:106–23. 27180913 10.1016/j.cpr.2016.04.011

[26] Faraone SV, Rostain AL, Blader J, Busch B, Childress AC, Connor DF, et al. Practitioner review: Emotional dysregulation in attention-deficit/hyperactivity disorder–implications for clinical recognition and intervention. J Child Psychol Psychiatry. 2019;60(2):133–50. 29624671 10.1111/jcpp.12899

[27] Christiansen H, Hirsch O, Albrecht B, Chavanon M-L. Attention-deficit/hyperactivity disorder (ADHD) and emotion regulation over the life span. Curr Psychiatry Rep. 2019;21:1–11. 30826879 10.1007/s11920-019-1003-6

[28] Beheshti A, Chavanon M-L, Christiansen H. Emotion dysregulation in adults with attention deficit hyperactivity disorder: a meta-analysis. BMC Psychiatry. 2020;20:1–11. 32164655 10.1186/s12888-020-2442-7 PMC7069054

[29] Eyre O, Langley K, Stringaris A, Leibenluft E, Collishaw S, Thapar A. Irritability in ADHD: Associations with depression liability. J Affect Disord. 2017;215:281–7. 28363151 10.1016/j.jad.2017.03.050 PMC5409953

[30] Sobanski E, Banaschewski T, Asherson P, Buitelaar J, Chen W, Franke B, et al. Emotional lability in children and adolescents with attention deficit/hyperactivity disorder (ADHD): clinical correlates and familial prevalence. J Child Psychol Psychiatry. 2010;51(8):915–23. 20132417 10.1111/j.1469-7610.2010.02217.x

[31] Seymour KE, Macatee R, Chronis-Tuscano A. Frustration tolerance in youth with ADHD. J Atten Disord. 2019;23(11):1229–39. 27282378 10.1177/1087054716653216 PMC6541529

[32] Althoff RR, Verhulst FC, Rettew DC, Hudziak JJ, van der Ende J. Adult outcomes of childhood dysregulation: a 14-year follow-up study. J Am Acad Child Adolesc Psychiatry. 2010;49(11):1105–16. 20970698 10.1016/j.jaac.2010.08.006 PMC2965164

[33] Biederman J, Spencer TJ, Petty C, Hyder LL, O’Connor KB, Surman CB, et al. Longitudinal course of deficient emotional self-regulation CBCL profile in youth with ADHD: prospective controlled study. Neuropsychiatr Dis Treat. 2012;8:267–76. 22848182 10.2147/NDT.S29670 PMC3404687

[34] Thompson RA. Emotion regulation: a theme in search of definition. Monogr Soc Res Child Dev. 1994;9:25–52. 7984164

[35] Gross JJ. Emotion regulation: current status and future prospects. Psychol Inquiry. 2015;26(1):1–26.

[36] Grosbras M-H, Ross PD, Belin P. Categorical emotion recognition from voice improves during childhood and adolescence. Sci Rep. 2018;8(1):14791. 30287837 10.1038/s41598-018-32868-3 PMC6172235

[37] Thomas LA, De Bellis MD, Graham R, LaBar KS. Development of emotional facial recognition in late childhood and adolescence. Dev Sci. 2007;10(5):547–58. 17683341 10.1111/j.1467-7687.2007.00614.x

[38] Brackett MA, Rivers SE, Shiffman S, Lerner N, Salovey P. Relating emotional abilities to social functioning: a comparison of self-report and performance measures of emotional intelligence. J Person Soc Psychol. 2006;91(4):780. 10.1037/0022-3514.91.4.780 17014299

[39] Aspan N, Bozsik C, Gadoros J, Nagy P, Inantsy-Pap J, Vida P, et al. Emotion recognition pattern in adolescent boys with attention-deficit/hyperactivity disorder. BioMed Res Int. 2014;2014(1):761340. 25110694 10.1155/2014/761340 PMC4119615

[40] Lievore R, Crisci G, Mammarella IC. Emotion recognition in children and adolescents with ASD and ADHD: a systematic review. Rev J Autism Dev Disord. 2023;8:1–31. 10.1007/s10803-025-07120-3 41252053

[41] Staff AI, Luman M, Van der Oord S, Bergwerff CE, van den Hoofdakker BJ, Oosterlaan J. Facial emotion recognition impairment predicts social and emotional problems in children with (subthreshold) ADHD. Eur Child Adolesc Psychiatry. 2021;8:1–13. 10.1007/s00787-020-01709-y PMC9142461 33415471

[42] McKay E, Cornish K, Kirk H. Impairments in emotion recognition and positive emotion regulation predict social difficulties in adolescent with ADHD. Clin Child Psychol Psychiatry. 2023;28(3):895–908. 36440882 10.1177/13591045221141770

[43] Nock MK, Wedig MM, Holmberg EB, Hooley JM. The emotion reactivity scale: development, evaluation, and relation to self-injurious thoughts and behaviors. Behav Therapy. 2008;39(2):107–16. 10.1016/j.beth.2007.05.005 18502244

[44] Dahl RE, Gunnar MR. Heightened stress responsiveness and emotional reactivity during pubertal maturation: Implications for psychopathology. Dev Psychopathol. 2009;21(1):1–6. 19144219 10.1017/S0954579409000017

[45] Carlo G, Crockett LJ, Wolff JM, Beal SJ. The role of emotional reactivity, self-regulation, and puberty in adolescents’ prosocial behaviors. Soc Dev. 2012;21(4):667–85. 28316370 10.1111/j.1467-9507.2012.00660.x PMC5356224

[46] McQuade JD, Breaux RP. Are elevations in ADHD symptoms associated with physiological reactivity and emotion dysregulation in children?. J Abnorm Child Psychol. 2017;45:1091–103. 10.1007/s10802-016-0227-8 27838892

[47] Jensen SA, Rosen L. Emotional reactivity in children with attention-deficit/hyperactivity disorder. J Atten Disord. 2004;8(2):53–61. 15801335 10.1177/108705470400800203

[48] Bunford N, Evans SW, Langberg JM. Emotion dysregulation is associated with social impairment among young adolescents with ADHD. J Atten Disord. 2018;22(1):66–82. 24681899 10.1177/1087054714527793

[49] Garnefski N, Kraaij V, Spinhoven P. Negative life events, cognitive emotion regulation and emotional problems. Person Indiv Diff. 2001;30(8):1311–27.

[50] Garnefski N, Legerstee J, Kraaij V, van Den Kommer T, Teerds J. Cognitive coping strategies and symptoms of depression and anxiety: a comparison between adolescents and adults. J Adolesc. 2002;25(6):603–11. 12490178 10.1006/jado.2002.0507

[51] Aldao A, Nolen-Hoeksema S. Specificity of cognitive emotion regulation strategies: a transdiagnostic examination. Behav Res Therapy. 2010;48(10):974–83. 10.1016/j.brat.2010.06.002 20591413

[52] Mayer JS, Brandt GA, Medda J, Basten U, Grimm O, Reif A, et al. Depressive symptoms in youth with ADHD: the role of impairments in cognitive emotion regulation. Eur Arch Psychiatry Clin Neurosci. 2022;272(5):793–806. 35107603 10.1007/s00406-022-01382-z PMC9279209

[53] Luo Y, Weibman D, Halperin JM, Li X. A review of heterogeneity in attention deficit/hyperactivity disorder (ADHD). Front Hum Neurosci. 2019;13:42. 30804772 10.3389/fnhum.2019.00042 PMC6378275

[54] Levy F, Hay DA, McSTEPHEN M, Wood C, Waldman I. Attention-deficit hyperactivity disorder: a category or a continuum? Genetic analysis of a large-scale twin study. J Am Acad Child Adolesc Psychiatry. 1997;36(6):737–44. 9183127 10.1097/00004583-199706000-00009

[55] Seymour KE, Chronis-Tuscano A, Iwamoto DK, Kurdziel G, MacPherson L. Emotion regulation mediates the association between ADHD and depressive symptoms in a community sample of youth. J Abnormal Child Psychol. 2014;42:611–21. 10.1007/s10802-013-9799-8 PMC4207628 24221724

[56] Caspi A, Houts RM, Belsky DW, Goldman-Mellor SJ, Harrington H, Israel S, et al. The p factor: one general psychopathology factor in the structure of psychiatric disorders? Clin Psychol Sci. 2014;2(2):119–37. 25360393 10.1177/2167702613497473 PMC4209412

[57] Kotov R, Cicero DC, Conway CC, DeYoung CG, Dombrovski A, Eaton NR, et al. The hierarchical taxonomy of psychopathology (HiTOP) in psychiatric practice and research. Psychol Med. 2022;52(9):1666–78. 35650658 10.1017/S0033291722001301

[58] Dell’Osso L, Lorenzi P, Carpita B. The neurodevelopmental continuum towards a neurodevelopmental gradient hypothesis. J Psychopathol. 2019;25(4):179–82.

[59] Sanchis-Sanchis A, Grau MD, Moliner A-R, Morales-Murillo CP. Effects of age and gender in emotion regulation of children and adolescents. Front Psychol. 2020;11:946. 32528367 10.3389/fpsyg.2020.00946 PMC7265134

[60] Rapee RM, Oar EL, Johnco CJ, Forbes MK, Fardouly J, Magson NR, et al. Adolescent development and risk for the onset of social-emotional disorders: a review and conceptual model. Behav Res Therapy. 2019;123:103501. 10.1016/j.brat.2019.103501 31733812

[61] Fedele DA, Lefler EK, Hartung CM, Canu WH. Sex differences in the manifestation of ADHD in emerging adults. J Atten Disord. 2012;16(2):109–17. 21173428 10.1177/1087054710374596

[62] Zimmermann P, Iwanski A. Emotion regulation from early adolescence to emerging adulthood and middle adulthood: age differences, gender differences, and emotion-specific developmental variations. Int J Behav Dev. 2014;38(2):182–94.

[63] Micoulaud-Franchi J-A, Weibel S, Weiss M, Gachet M, Guichard K, Bioulac S, et al. Validation of the French version of the Weiss functional impairment rating scale–self-report in a large cohort of adult patients with ADHD. J Atten Disord. 2019;23(10):1148–59. 30191748 10.1177/1087054718797434

[64] Canu WH, Hartung CM, Stevens AE, Lefler EK. Psychometric properties of the Weiss Functional Impairment Rating Scale: evidence for utility in research, assessment, and treatment of ADHD in emerging adults. J Atten Disord. 2020;24(12):1648–60. 27481918 10.1177/1087054716661421

[65] Hadianfard H, Kiani B, Weiss MD. Psychometric properties of the Persian version of the Weiss Functional Impairment Rating Scale–Self-report form in Iranian adolescents. J Atten Disord. 2019;23(13):1600–9. 29099238 10.1177/1087054717738084

[66] Kessler RC, Adler L, Ames M, Demler O, Faraone S, Hiripi E, et al. The World Health Organization Adult ADHD self-report scale (ASRS): a short screening scale for use in the general population. Psychol Med. 2005;35(2):245–56. 15841682 10.1017/s0033291704002892

[67] Green JG, DeYoung G, Wogan ME, Wolf EJ, Lane KL, Adler LA. Evidence for the reliability and preliminary validity of the adult ADHD self-report scale v1.1 (ASRS v1.1) screener in an adolescent community sample. Int J Methods Psychiatr Res. 2019;28(1):1751. 10.1002/mpr.1751 PMC6877133 30407687

[68] Sonnby K, Skordas K, Olofsdotter S, Vadlin S, Nilsson KW, Ramklint M. Validation of the World Health Organization adult ADHD self-report scale for adolescents. Nordic J Psychiatry. 2015;69(3):216–23. 10.3109/08039488.2014.968203 25348323

[69] Robertson IH, Manly T, Andrade J, Baddeley BT, Yiend J. Oops!‘: performance correlates of everyday attentional failures in traumatic brain injured and normal subjects. Neuropsychologia. 1997;35(6):747–58. 9204482 10.1016/s0028-3932(97)00015-8

[70] Schlegel K, Scherer KR. Introducing a short version of the Geneva Emotion Recognition Test (GERT-S): psychometric properties and construct validation. Behav Res Methods. 2016;48:1383–92. 26416137 10.3758/s13428-015-0646-4

[71] Lannoy S, Heeren A, Rochat L, Rossignol M, Van der Linden M, Billieux J. Is there an all-embracing construct of emotion reactivity? Adaptation and validation of the emotion reactivity scale among a French-speaking community sample. Comprehens Psychiatry. 2014;55(8):1960–7. 10.1016/j.comppsych.2014.07.023 25176623

[72] Jermann F, Van der Linden M, d’Acremont M, Zermatten A. Cognitive emotion regulation questionnaire (CERQ). Eur J Psychol Assess. 2006;22(2):126–31.

[73] McLaughlin KA, Hatzenbuehler ML, Mennin DS, Nolen-Hoeksema S. Emotion dysregulation and adolescent psychopathology: a prospective study. Behav Res Therapy. 2011;49(9):544–54. 10.1016/j.brat.2011.06.003 PMC3153591 21718967

[74] Schreiber LR, Grant JE, Odlaug BL. Emotion regulation and impulsivity in young adults. J Psychiatr Res. 2012;46(5):651–8. 22385661 10.1016/j.jpsychires.2012.02.005 PMC3334448

[75] Sánchez M, Lavigne R, Romero JF, Elósegui E. Emotion regulation in participants diagnosed with attention deficit hyperactivity disorder, before and after an emotion regulation intervention. Front Psychol. 2019;10:1092. 31178779 10.3389/fpsyg.2019.01092 PMC6543912

[76] Spear LP. The adolescent brain and age-related behavioral manifestations. Neurosci Biobehav Rev. 2000;24(4):417–63. 10817843 10.1016/s0149-7634(00)00014-2

[77] Gross JJ. Emotion regulation: affective, cognitive, and social consequences. Psychophysiology. 2002;39(3):281–91. 12212647 10.1017/s0048577201393198

[78] Manju H. Cognitive regulation of emotion and quality of life. J Psychosoc Res. 2017;12(1):1.

[79] Bodalski EA, Knouse LE, Kovalev D. Adult ADHD, emotion dysregulation, and functional outcomes: Examining the role of emotion regulation strategies. J Psychopathol Behav Assess. 2019;41:81–92.

[80] Maynard ML, Quenneville S, Hinves K, Talwar V, Bosacki SL. Interconnections between emotion recognition, self-processes and psychological well-being in adolescents. Adolescents. 2022;3(1):41–59.

[81] Mathews A, May J, Mogg K, Eysenck M. Attentional bias in anxiety: selective search or defective filtering? J Abnorm Psychol. 1990;99(2):166. 2348010 10.1037//0021-843x.99.2.166

[82] Mogg K, Garner M, Bradley BP. Anxiety and orienting of gaze to angry and fearful faces. Bio Psychol. 2007;76(3):163–9. 17764810 10.1016/j.biopsycho.2007.07.005 PMC2075532

[83] Hadianfard H, Kiani B, Weiss MD. Study of functional impairment in students of elementary and secondary public schools in Iran. J Can Acad Child Adolesc Psychiatry. 2021;30(2):68. 33953759 PMC8056958

[84] De Rossi P, D’Aiello B, Pretelli I, Menghini D, Di Vara S, Vicari S. Age-related clinical characteristics of children and adolescents with ADHD. Front Psychiatry. 2023;14:1069934. 36778635 10.3389/fpsyt.2023.1069934 PMC9911799

[85] Crone EA, Green KH, van de Groep IH, van der Cruijsen R. A neurocognitive model of self-concept development in adolescence. Ann Rev Dev Psychol. 2022;4:273–95.

[86] Bleidorn W, Arslan RC, Denissen JJ, Rentfrow PJ, Gebauer JE, Potter J, et al. Age and gender differences in self-esteem—A cross-cultural window. J Person Soc Psychol. 2016;111(3):396. 10.1037/pspp0000078 26692356

[87] Meeus W. Adolescent psychosocial development: a review of longitudinal models and research. Dev Psychol. 2016;52(12):1969. 27893243 10.1037/dev0000243

[88] Van der Graaff J, Branje S, De Wied M, Hawk S, Van Lier P, Meeus W. Perspective taking and empathic concern in adolescence: gender differences in developmental changes. Dev Psychol. 2014;50(3):881. 24040846 10.1037/a0034325

[89] Poznyak E, Morosan L, Perroud N, Speranza M, Badoud D, Debbané M. Roles of age, gender and psychological difficulties in adolescent mentalizing. J Adolesc. 2019;74:120–9. 31202040 10.1016/j.adolescence.2019.06.007

[90] Keulers EH, Evers EA, Stiers P, Jolles J. Age, sex, and pubertal phase influence mentalizing about emotions and actions in adolescents. Dev Neuropsychol. 2010;35(5):555–69. 20721775 10.1080/87565641.2010.494920

[91] Burnette CB, Kwitowski MA, Mazzeo SE. “I don’t need people to tell me I’m pretty on social media:” a qualitative study of social media and body image in early adolescent girls. Body Image. 2017;23:114–25. 28965052 10.1016/j.bodyim.2017.09.001

[92] Mittal S, Verma P, Jain N, Khatter S, Juyal A. Gender based variation in cognitive functions in adolescent subjects. Ann Neurosci. 2012;19(4):165. 25205992 10.5214/ans.0972.7531.190406 PMC4117054

[93] Lyons KE, Zelazo PD. Monitoring, metacognition, and executive function: elucidating the role of self-reflection in the development of self-regulation. Adv Child Dev Behav. 2011;40:379–412. 21887967 10.1016/b978-0-12-386491-8.00010-4

[94] Taylor SJ, Barker LA, Heavey L, McHale S. The longitudinal development of social and executive functions in late adolescence and early adulthood. Front Behav Neurosci. 2015;9:252. 26441579 10.3389/fnbeh.2015.00252 PMC4569858

[95] Rafi H, Murray R, Delavari F, Perroud N, Vuilleumier P, Debbané M, et al. Neural basis of internal attention in adults with pure and comorbid ADHD. J Atten Disord. 2023;27(4):423–36. 36635890 10.1177/10870547221147546 PMC13035225

[96] Rescorla LA, Ginzburg S, Achenbach TM, Ivanova MY, Almqvist F, Begovac I, et al. Cross-informant agreement between parent-reported and adolescent self-reported problems in 25 societies. J Clin Child Adolesc Psychol. 2013;42(2):262–73. 23009025 10.1080/15374416.2012.717870

